# Membrane Activity and Viroporin Assembly for the SARS-CoV-2 E Protein Are Regulated by Cholesterol

**DOI:** 10.3390/biom14091061

**Published:** 2024-08-26

**Authors:** Marta V. Volovik, Zaret G. Denieva, Polina K. Gifer, Maria A. Rakitina, Oleg V. Batishchev

**Affiliations:** 1 Laboratory of Bioelectrochemistry, A.N. Frumkin Institute of Physical Chemistry and Electrochemistry, Russian Academy of Sciences, 31/4 Leninskiy Prospekt, 119071 Moscow, Russia; marta.volovik@phystech.edu (M.V.V.); zaret03@mail.ru (Z.G.D.); gifer.pk@phystech.edu (P.K.G.); 2 N.I. Pirogov Russian National Research Medical University of the Ministry of Health of the Russian Federation, 1 Ostrovityanova Street, 117997 Moscow, Russia

**Keywords:** SARS-CoV-2 E protein, viroporin, H3 peptide, lipid membrane, cholesterol, vesicles, defects, pores, atomic force microscopy, fluorescence confocal microscopy, patch-clamp

## Abstract

The SARS-CoV-2 E protein is an enigmatic viral structural protein with reported viroporin activity associated with the acute respiratory symptoms of COVID-19, as well as the ability to deform cell membranes for viral budding. Like many viroporins, the E protein is thought to oligomerize with a well-defined stoichiometry. However, attempts to determine the structure of the protein complex have yielded inconclusive results, suggesting several possible oligomers, ranging from dimers to pentamers. Here, we combined patch-clamp, confocal fluorescence microscopy on giant unilamellar vesicles, and atomic force microscopy to show that E protein can exhibit two modes of membrane activity depending on membrane lipid composition. In the absence or the presence of a low content of cholesterol, the protein forms short-living transient pores, which are seen as semi-transmembrane defects in a membrane by atomic force microscopy. Approximately 30 mol% cholesterol is a threshold for the transition to the second mode of conductance, which could be a stable pentameric channel penetrating the entire lipid bilayer. Therefore, the E-protein has at least two different types of activity on membrane permeabilization, which are regulated by the amount of cholesterol in the membrane lipid composition and could be associated with different types of protein oligomers.

## 1. Introduction

The causative agent of COVID-19 is the SARS-CoV-2 coronavirus, the third coronavirus after SARS-CoV and MERS-CoV, being the reason for severe respiratory infections in humans and the only one resulting in a devastating pandemic. Coronaviruses are a class of positive-strand-RNA-enveloped viruses having a lipid membrane over a protein capsid protecting the viral genome. The SARS-CoV-2 genome encodes four main structural proteins: spike (S), membrane (M), envelope (E), and nucleocapsid (N) [1]. The S protein mediates binding of the virus to host cell ACE2 receptors, resulting in virus penetration into the cell. The M protein determines the shape of the viral envelope. The N protein binds to the RNA genome and is responsible for its packaging. The E protein is the smallest (75 amino acid residues), highly conserved, but least understood protein of SARS-CoV-2 [2]. Its primary sequence is 95% identical to the E protein of SARS-CoV and 36% identical to the one of MERS-CoV [3], thus suggesting a close similarity between the SARS-CoV and SARS-CoV-2 E proteins. Being involved in the assembly and budding of progeny virions, it is crucial for the formation of infectious virions [4,5,6]. On the other hand, some studies have reported ion channel activity of the E protein, placing it amongst viroporins of the class I [7,8,9,10]. Finally, it stimulates inflammation inside the cell, yielding a cytokine storm and the acute respiratory syndrome which gives the name to this class of coronaviruses [11].

The E protein of SARS-CoV-2 comprises three main structural parts: a long amphipathic C-terminus facing the cell cytoplasm, a short hydrophilic N-terminus, and a highly hydrophobic transmembrane domain [12]. Additionally, the E protein undergoes co- and post-translational modifications resulting in its palmitoylation and glycosylation of the C-terminal part [13,14]. Possibly, glycosylation may protect the protein from the host immune response [15]. However, recent in vivo studies suggest that the majority of E proteins are probably not glycosylated [3]. Palmitoylation is known, e.g., for the cytoplasmic tail of the influenza A virus haemagglutinin, and is considered to be responsible for the protein–protein interactions between the viral structural proteins [16]. In the case of the Gag polyprotein of the human immunodeficiency virus, acylation is not crucial for the membrane activity of this protein [17]. Therefore, the necessity of these modifications of the SARS-CoV-2 E protein is still unclear [14].

The C-terminal part of the E protein contains the PDZ domain-binding motif (PBM), which binds the viral M protein, enhancing viral budding [14]. The PBM is also able to bind different cellular proteins like inflammatory cytokines, responsible for the pathogenic cytokine storm associated with SARS-CoV infection [18]. Thus, the C-terminal part of the E protein is believed to be a significant virulence factor [19]. In [20], the authors suggest a leading role of the C-terminus in generating membrane curvature for the budding on newly assembled viruses at the ultimate stage of infection. According to the authors, the E protein primes the membrane for the further budding by deforming it with its amphipathic C-terminal helix. However, other studies suggest that the membrane curvature itself is the first violin in the orchestra [21]. Based on structural studies of the E protein, the authors hypothesize that the C-terminal part of the E protein adopts a β-sheet conformation, transforming into an α-helix only at highly curved regions of the endoplasmic reticulum–Golgi intermediate compartment (ERGIC) lumen. This is in line with the results of [22], where the authors show that the E protein itself keeps the membrane flat and the membrane curvature is generated by the M-protein, which further binds the E protein. However, the absence of the E protein significantly reduces virion formation [23]. Therefore, the role of the C-terminal part of the E protein in SARS-CoV-2 pathogenesis is still under debate.

The N-terminal and transmembrane parts of the protein are believed to be responsible for its ion channel activity and are proposed to have identical mechanisms of action to other viral ion channels, viroporins [24]. This functionality is based on the paradigm of the protein oligomerization into pentameric channels with low cation selectivity [25,26,27,28]. Viroporins represent a group of virus-encoded proteins that share the common function of forming membrane channels in infected host cells [8,9,10,29,30]. These channels play a crucial role in various stages of the viral life cycle, including viral entry, replication, and assembly of viral particles and their release from the host cell. Viroporins were originally identified as a new family of viral proteins due to their ability to alter membrane permeability to ions or other small molecules [31]. The structure of the viroporins typically includes a relatively small number of amino acid residues, ranging from 60 to 120, and at least one transmembrane hydrophobic domain with an amphipathic helix [8,32,33]. The amphipathic helix within the transmembrane domain consists of hydrophobic and hydrophilic residues arranged in a way that allows viroporin to insert into the host cell membrane and form ion channels. The structure of the channels and the impact on the virus life cycle can vary significantly based on the sequence and characteristics of the specific viroporin [9,34]. Generally, they oligomerize and form ion-conducting channels, disrupting ion homeostasis and altering cellular processes [35]. This disruption of membrane integrity can promote viral replication, and modulate cellular signaling and host immune responses [8]. Viroporins may also alter membrane curvature or promote membrane scission, necessary for viral particle release [36]. Several viroporins are known to be involved in the formation of double-membrane vesicles (DMVs), which are distinct structures separate from the endoplasmic reticulum (ER) [37,38,39,40]. These DMVs serve as specialized compartments for the replication and assembly of coronaviruses.

It is still not clear why SARS-CoV-2 requires such an ion channel. The well-known example of the M2 proton channel from the influenza A virus is undoubtedly connected with the endocytic pathway of this virus and necessary to acidify the viral interior for RNP release [41]. At the ultimate stage of infection, it participates in scission of the nascent virion from the plasma membrane of the infected cell by its amphipathic helices [42]. Thus, the M2 protein exhibits ion channel activity, necessary at the entry stage, and has membrane-deforming properties at the budding stage. Ion channels formed by the E protein have cationic selectivity in negatively charged membranes, while they are almost non-selective in neutral membranes at pH 6 [7,43]. In [44], the authors report an even better conductance of the SARS-CoV-2 viroporin for divalent cations compared to Na^+^ or K^+^. Moreover, just a little fraction of the E protein transfers from the ER, ERGIC, and Golgi to the virion envelope. Therefore, the role of this protein at different stages of the viral lifecycle is not clear.

Recent studies contradict the idea of the pentameric channel demonstrating other possible types of the E protein assembly, such as dimers [45,46]. It is noteworthy that even two amphipathic molecules are enough to make an ion-conducting defect in the lipid membrane [47]. This fact also emphasizes the role of the lipid environment, especially charged lipid and cholesterol, for the assembly of the E protein monomers and the ion channel activity of its oligomers [7,20,21,48]. The exact route of the SARS-CoV-2 budding from the infected cell is still unclear [49]. Therefore, the E protein on this route may interact with different cellular membrane structures, from ERGIC to the plasma membrane, the lipid compositions of which significantly vary. For example, the cholesterol content of the ERGIC compartment is about 15 mol% [50], while for the plasma membrane it reaches 40 mol% [51]. Moreover, different types of the protein oligomers may induce different structures of membrane pores or channels. Not only the transmembrane domain but also the juxtamembrane regions of the E protein might be involved in the protein-induced membrane conductance.

Therefore, in the present study, we decided to clarify the role of the lipid environment and distinct parts of the SARS-CoV-2 E protein in both the membrane curvature generation and the ion-conducting activity of the protein. For this purpose, we used recombinant SARS-CoV-2 E protein without glycosylation and palmitoylation, as well as separately synthesized H3 helix from its C-terminal part (a.a. 53–68), with varying content of charged lipids and cholesterol in different model lipid membranes. We utilized fluorescent confocal microscopy on giant unilamellar vesicles (GUVs) to elucidate the ability of the E protein and its H3 helix to deform the membrane and induce dye leakage. This method was combined with patch-clamp measurements of the viroporin activity of the protein and its C-terminal part to better understand the structure and lifetime of the formed pores and/or ion channels. Finally, we applied atomic force microscopy to show at the molecular level the membrane activity of the protein and its cytoplasmic juxtamembrane part.

## 2. Materials and Methods

### 2.1. Materials

The following lipids were purchased from Avanti Polar Lipids (Alabaster, AL, USA) and were used without further purification: 1-palmitoyl-2-oleoyl-glycero-3-phosphocholine (POPC), 1-palmitoyl-2-oleoyl-sn-glycero-3-phospho-L-serine (sodium salt) (POPS), 1,2-dioleoyl-*sn*-glycero-3-phosphoethanolamine (DOPE), and cholesterol (Chol). The labeled lipid 1,2-dioleoyl-sn-glycero-3-phosphoethanolamine-N-lissamine rhodamine B sulfonyl (Rho-DOPE) from the same vendor was used for the fluorescence microscopy. Lipid solutions were prepared using chloroform, squalane, octane, and decane (all from Sigma Aldrich, Saint-Louis, MO, USA, purity > 99%).

Anhydrous glucose and sucrose (both from SERVA, Heidelberg, Germany), NaCl (Sigma-Aldrich, Saint-Louis, MO, USA), KCl (Sigma-Aldrich, Saint-Louis, MO, USA), HEPES (Helicon, Moscow, Russia), phosphate buffer (PBS) in tablets (Helicon, Moscow, Russia), bovine serum albumin (BSA) (Sigma-Aldrich, Saint-Louis, MO, USA, purity > 98%), calcein (Sigma-Aldrich, Saint-Louis, MO, USA), recombinant SARS-CoV-2 E protein with SUMO-1 tag (Elabscience, Houston, TX, USA, purity > 90%, SDS-PAGE), hereinafter referred as the E protein, and recombinant human SUMO-1 (FineTest Biotech Inc., Boulder, CO, USA, purity > 95%, SDS-PAGE) were used without further purification.

The H3 peptide of the E protein (^53^KPSFYVYSRVKNLNSS^68^) was obtained by solid-phase synthesis on Rink Amide resin. The synthesis was carried out with microwave irradiation of the reaction mixture using the Fmoc strategy. The purity of the peptide (>97% pure) was accessed by HPLC and mass spectrometry (Figure S1). The MW of the peptide is 1889 Da.

### 2.2. Formation of Giant Unilamellar Vesicles (GUVs)

GUVs were prepared by the classical method of electroformation [52]. Lipid stock solutions of 10 mg/mL lipid in chloroform were mixed in a 0.5 mL Eppendorf vial to obtain the compositions of lipids in different ratios, containing 0.5 mol% of fluorescent dye Rho-DOPE (0.01 g/L, total lipid). A volume of 3 μL of a 5 mg/mL lipid solution in chloroform was applied to each of two pre-cleaned ITO glass slides (2.5 × 2.5 cm^2^) and dried under an argon stream for 1 min. The glass slides were then placed in a polypropylene chamber at a distance of 4 mm from each other. The chamber was filled with the swelling buffer (SB; 270 mM sucrose, 2 mM KCl, 10 mM HEPES, pH 6.0, and 4 mM calcein), sealed with Parafilm and placed in a thermostat at 55 °C. A sinusoidal signal with an incremental amplitude of 2 V and a frequency of 11.2 Hz was then applied to the chamber using a function generator. The GUVs were allowed to grow for 3 h, collected in Eppendorf vials, and stored at 4 °C for no longer than two days. The osmolarity of the GUV suspension was controlled before each measurement using an osmometer (Osmomat 030, Gonotec GmbH, Berlin, Germany). The osmolarity of the working buffer 1, WB1 (PBS, 30 mM NaCl, pH 6.0) was adjusted with glucose to maintain isosmotic conditions.

### 2.3. Confocal Fluorescence Microscopy

The GUVs were imaged using a laser scanning Ti2 Eclipse confocal fluorescence microscope (Nikon, Tokyo, Japan) with a 60× magnification objective using the single-GUV method [53]. For the experiments, 1 mg/mL BSA in a water solution was applied to glass coverslips for one hour. Further, they were washed with Milli-Q (MilliPore, Direct-Q 3UV system, Burlington, MA, USA) water and air dried. This pre-treatment prevents the GUVs from flattening and rupture when they are in contact with the glass surface [54]. A volume of 5 μL of GUVs in the swelling buffer was diluted in 200 μL of the working buffer 1. This droplet was placed on a glass coverslip and equilibrated for 5 min. All experiments were performed at room temperature and in isosmotic conditions. Lasers at 488 nm and 561 nm were used for calcein and Rho-DOPE fluorescence excitation, respectively. We selected a GUV with a diameter of 5 μm or above and focused on its equatorial plane. To ensure that there were no protrusions or small vesicles inside the vesicle, z-stack images were collected. The procedure was repeated three times within 5 min prior to the protein addition. We analyzed 3–5 GUVs from different preparations in each experiment. SARS-CoV-2 E protein, H3 peptide, as well as control SUMO-1 were added to the selected GUV through a glass micropipette (with a tip of 1 μm in diameter) placed in the vicinity of the selected GUV (Figure S2). For the E protein and SUMO-1 the concentration was 1 μM, while for H3 peptide it was 20 μM. The distance between the GUV and the tip of the micropipette was 10 μm, and a small positive pressure was applied to induce a flow of the protein solution from the pipette.

The obtained images were processed using the NIS Elements (Nikon, Tokyo, Japan) and ImageJ version 1.54j (NIH, Bethesda, DC, USA) software.

### 2.4. Calcein Leakage Assay

The process of pore formation by the E protein, its H3 peptide, and SUMO-1 was investigated by the change in the GUV fluorescence intensity due to calcein leakage. A complete (100%) leakage of calcein was performed by the addition of Triton X-100 in a final concentration of 0.07% (*v*/*v*). The average intensity per GUV was estimated and normalized to the intensity of the intact vesicle at the initial time point (0 min). The release of fluorescent dye from the GUV was measured as follows:

$$leakage\;\left(\%\right)=\frac{I_{m}-I_{0}}{I_{100}-I_{0}}\times 100$$

where *I_m_* is the measured fluorescence intensity; *I*_0_ and *I*_100_ are the fluorescence intensities at time zero and after the addition of Triton X-100, respectively.

In the case of intact GUVs before protein addition (negative control), there was almost no calcein leakage (less than 5%) within 30 min (Figure S3).

### 2.5. Formation of Planar Bilayer Lipid Membranes (BLMs)

Lipid stock solutions of 10 mg/mL lipid in chloroform were mixed in a 1.5 mL Eppendorf vial to obtain the lipid compositions in different ratios. The solvent was removed by drying under an argon stream for 30 min, and then, the mixture was dissolved in squalane at a concentration of 20 mg/mL. Bilayer lipid membranes were formed by the Mueller–Rudin technique [55] on a 100 × 100 µm^2^ cooper mesh placed in a Petri dish. The mesh was preliminarily covered with 0.5 µL of the lipid solution in a 1:1 (*v*/*v*) mixture of octane and decane with a concentration of 10 mg/mL, followed by drying under argon to form a meniscus. The Petri dish was filled with WB1. The mesh was painted with the lipid solution in squalane, and BLMs were formed spontaneously.

### 2.6. Patch-Clamp Measurements

Measurements of the electrical current through the membrane were performed using a patch-clamp amplifier (HEKA EPC-8, Lambrecht, Germany) in a voltage-clamp mode with a pair of Ag/AgCl electrodes. The ground electrode was placed in the WB1 in a Petri dish, while the measuring one was placed in a glass micropipette filled with the same working buffer. The filter frequency of the low-pass filter of the patch-clamp amplifier was 5 kHz. The data acquisition frequency was 10 kHz. No software filters were used. After establishing a tight contact between the pipette and the BLM, a constant voltage of +100 mV was applied to the electrodes, and the current through the membrane was measured. A tight contact provided reliable electrical insulation of the fixed BLM patch from the rest of the membrane and the external solution. This system allowed us to monitor membrane conductance changes upon addition of the protein.

To monitor the pore formation by the proteins or peptide, the micropipette was filled with the corresponding substance dissolved in the WB1 at a concentration of 1 µM (for H3 peptide it was 20 μM). In each experiment, the membrane conductance was recorded for 10–30 min, and a total of 5 experiments was performed for each lipid mixture and the added substance.

To analyze the pore-forming activity of SARS-CoV-2 E protein, its H3 peptide, and SUMO-1, we calculated the number of experiments with conductance change for each min of the recording and divided it by the total number of experiments. The results of the calculations were presented as the relative occurrence of conductance changes. We used the definition of conducting events from [56], where the authors analyze different types of membrane conductance. Based on this work, we classified the patch-clamp signals into three types: spike, multi-level, and square-top. The spike signal is the fastest event, with a characteristic time of tens of milliseconds, corresponding to some short-living membrane pore. The multi-level shape represents a complex set of a broad spectrum of signals with varying lifetimes and amplitudes. Square-top signals have a step-like shape, with the almost constant conductance corresponding to steady-state channel-like structures. The percentage of conducting event types (spike, multi-level, or square-top) was estimated as the number of signals of the corresponding type during the total time of all experiments divided by the total amount of all signal types and multiplied by 100%. A conducting event was considered reliable if its amplitude was greater than twice the noise level.

### 2.7. Formation of Small Liposomes

To obtain small liposomes, lipids were dissolved in chloroform to the final concentration of 10 g/L. To prepare lipid films all components were combined in a given molar ratio in a round-bottom glass flask. This mixture in the flask was rotary evaporated under vacuum at a temperature of 37 °C for 40 min in total. The dried film was then dissolved in the working buffer 2 (WB2) (100 mM NaCl, 10 mM HEPES, pH 6.0) to the final concentration of 0.5 g/L. To form small vesicles, the lipid suspension in the flask was sonicated for 20 min at 55 °C. The vesicle suspension was utilized immediately after preparation.

### 2.8. Atomic Force Microscopy

AFM experiments were performed with a Multimode Nanoscope V (Bruker, Billerica, MA, USA) setup using an electrochemical fluid cell. All experiments were performed at room temperature in buffer solution. For scanning we used ultra sharp Silicon Tip on Nitride Lever (SNL-10) cantilevers with a nominal spring constant of 0.06 N/m and a tip radius of approximately 2 nm (Bruker, Billerica, MA, United States). The images were scanned at a dimension of 3 × 3 μm^2^. The resulting images were processed in the WSxM 5.0 software [57]. In the experiments, a 150 μL droplet of the pre-heated liposome suspension was placed on a freshly cleaved mica and incubated for 25 min at room temperature. Further vesicle rupture provided a supported lipid bilayer formation on the mica surface. Then, the sample was washed 7 times with WB2 and put into the fluid cell for further AFM imaging. All AFM experiments were performed in liquid in a way that the lipid bilayer was always covered with the buffer solution. To investigate the interaction of the SARS-CoV-2 E protein, its H3 peptide, and SUMO-1 with the supported bilayers, the protein or peptide stock solution was diluted with the WB2 to a final concentration of 100 nM. For the experiments, 100 μL of the solution was used. Each experiment was repeated at least 3 times with different sample preparations.

## 3. Results

### 3.1. E Protein but Not Its H3 Peptide Deform GUVs with Charged Lipids and High Cholesterol Content

**Figure 1:**
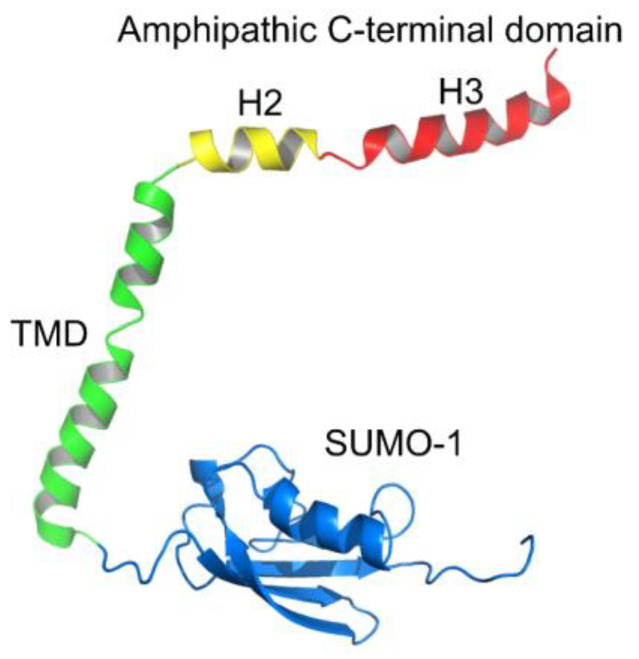
Schematic structure of the SARS-CoV-2 E protein monomer with its H3 peptide and SUMO-1 N-terminal tag. The structure was combined from the TMD structure obtained by NMR (PDB 7K3G [26]), structures of the H2 and H3 helices predicted in [20], and SUMO-1 structure (PDB 1A5R).

First, we decided to study the ability of the SARS-CoV-2 E protein and its H3 peptide (see Figure 1) to deform the membrane with different membrane lipid compositions.

POPC:POPS:DOPE:Chol = 44:17:24:15 mol% (herein referred to as mixture A);

POPC:POPS:DOPE = 59:17:24 by mol% (herein referred to as mixture B);

POPC:POPS:DOPE:Chol = 29:17:24:30 by mol% (herein referred to as mixture C);

POPC:POPS:DOPE:Chol = 19:17:24:40 by mol% (herein referred to as mixture D).

POPC:DOPE:Chol = 36:24:40 mol% herein referred to as mixture E).

Mixture A represents the lipid composition of the ERGIC membrane with only the exchange of POPE with a high-phase-transition temperature by DOPE [50]. Mixtures B–D represent varying cholesterol contents, from 0 to 40 mol%. The highest cholesterol content of 40 mol% is typical for the plasma membrane [51]. Mixture E is free of charged lipids but contains a maximum amount of 40 mol% cholesterol.

**Figure 2:**
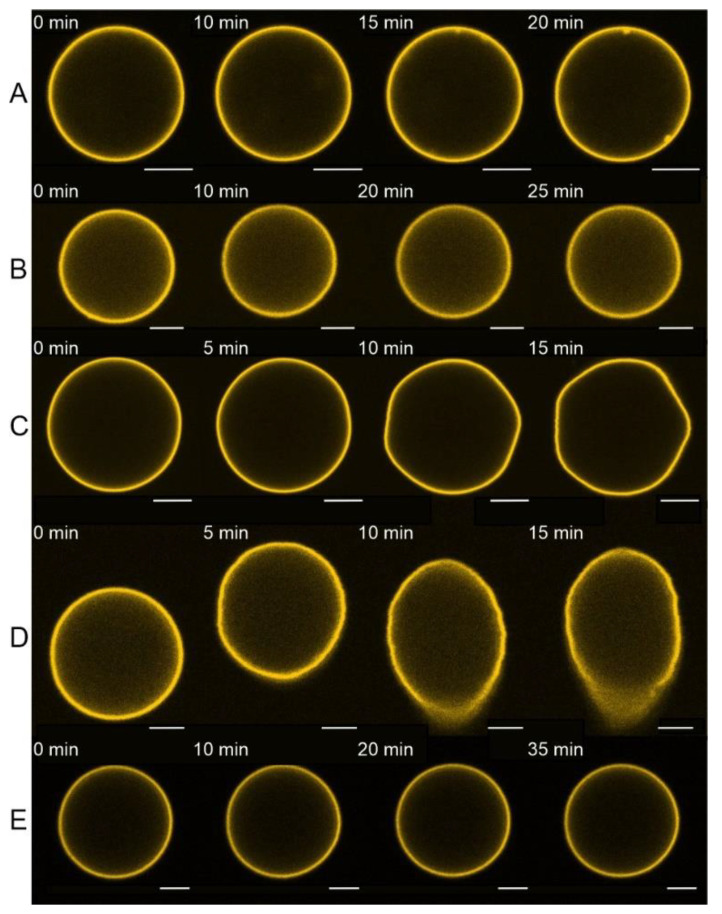
Typical fluorescence images of deformations of the GUV after the addition of 1 μM of the E protein in the vicinity of the GUV from lipid mixtures (**A**–**E**). The GUVs were labeled with 0.5 mol% of fluorescent dye Rho-DOPE. The numbers above each GUV indicate the time in min from the start of the protein addition. Scale bar is 10 µm.

GUVs were added to the glucose-equilibrated WB1 to create isosmotic conditions with the swelling buffer inside the GUVs. After selecting the GUV (see Materials and Methods for details), the E protein was added through a glass micropipette placed in the vicinity of the GUV. In cases of GUVs from mixtures A and B, we did not observe any changes in the vesicle shape for over 20 min (Figure 2). We started to observe deformations of the GUV in a threshold manner starting with mixture C, containing 30 mol% cholesterol. The strongest effect was observed for mixture D, with 40 mol% cholesterol. This may indicate an appearance of significant membrane leakage related to membrane reorganization during pore formation at high cholesterol content. However, when charged lipids were removed from this lipid composition (mixture E in Figure 2), no deformation was observed. It is noteworthy that we did not see any signs of formation of DMVs for any of the lipid mixtures studied.

The E protein itself is insoluble in water because of its highly hydrophobic transmembrane domain. Therefore, we used a construct of the protein with a SUMO-1 N-terminal tag, which makes the E protein soluble in water and the working buffers. To test the effect of SUMO-1 on the GUV shape, we performed experiments with solely SUMO-1 in the same molar concentration as for the construct with the E protein, for lipid mixture D. When the SUMO-1 protein was added to the GUVs of mixture D, filamentous protrusions formed inside them (see Supplementary Material, Figure S4A). The adsorption of isolated SUMO-1 protein appears to lead to lipid condensation in the outer monolayer of the GUV membrane via an electrostatic mechanism. This leads to an imbalance in the area of the outer and inner monolayers of the vesicle membrane, with the excess area on the inner monolayer being shed in the form of various membrane protrusions. We have previously observed such processes for various membrane proteins, including BSA and the matrix protein M1 of the influenza A virus [58]. However, no changes in the membrane shape similar to those observed for the construct of the E protein with SUMO-1 were detected. This might indicate that in the presence of the E protein, SUMO-1 neither affects the membrane shape nor causes lipid perturbations, unlike the isolated protein.

**Figure 3:**
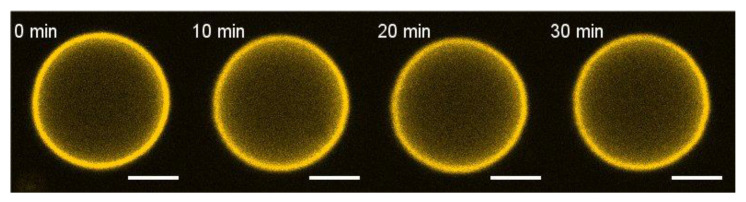
Typical fluorescence images of deformations of a GUV after the addition of 20 μM of the H3 peptide in the vicinity of the GUV from lipid mixture D. The GUVs were labeled with 0.5 mol% of fluorescent dye Rho-DOPE. The numbers above each GUV image indicate the time in min from the start of the peptide addition. Scale bar is 10 µm.

Since mixture D showed the most pronounced effect, we performed experiments with the H3 peptide using this lipid composition of the GUV. No changes in GUV shape were detected for even 20 μM of the peptide (Figure 3).

### 3.2. E Protein Induces Calcein Leakage from GUVs with Charged Lipids

**Figure 4:**
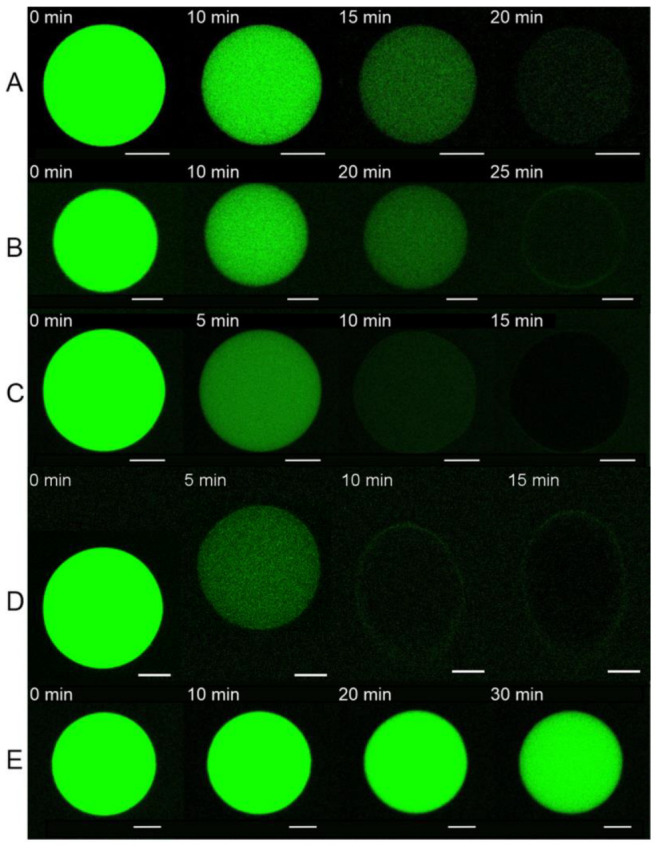
Typical fluorescence images of the calcein leakage from the GUVs from lipid mixtures (**A**–**E**) after the addition of 1 μM E protein. The numbers above each GUV indicate the time in min from the start of the protein addition. Scale bar is 10 µm.

**Figure 5:**
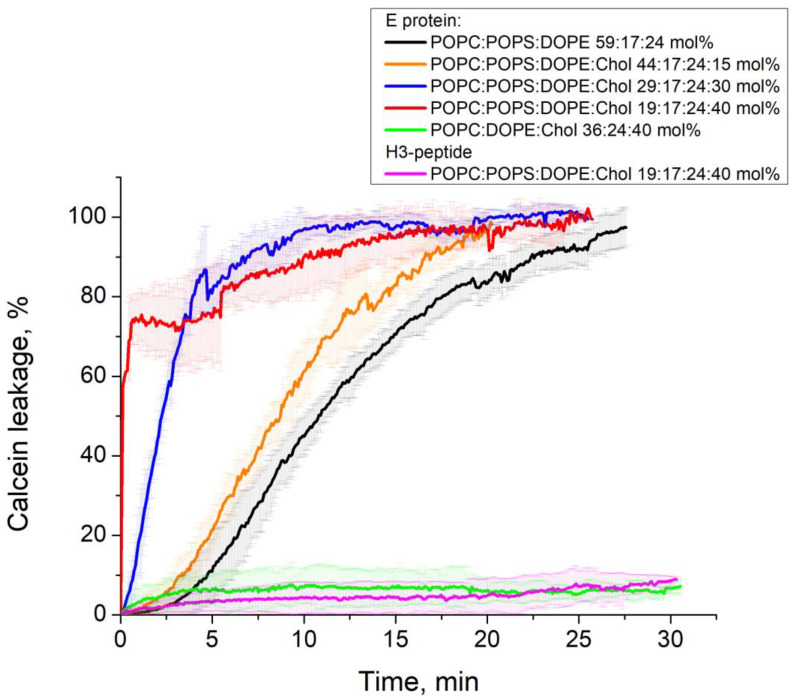
Typical kinetics of fluorescence intensity changes inside the GUV after the addition of 1 μM E protein in the vicinity of the GUVs from lipid mixture A (orange), mixture B (black), mixture C (blue), mixture D (red), and mixture E (green); and after the addition of 20 μM H3 peptide in the vicinity of the GUVs from lipid mixture D (pink). The results are summarized from 3 experiments of 30 min each for each lipid mixture (error bars for each lipid mixture are shown as SD in corresponding translucent color).

**Figure 6:**
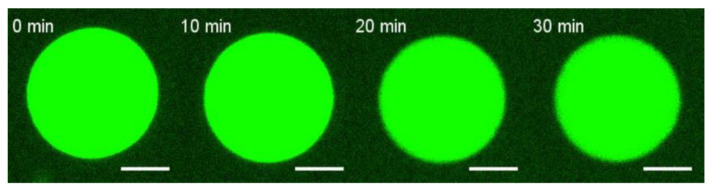
Typical fluorescence images of the calcein leakage from the GUVs from lipid mixture D after the addition of 20 μM H3 peptide. The numbers above each GUV indicate the time in min from the start of the protein addition. Scale bar is 10 µm.

To test the ability of the E protein and its H3 peptide to induce leakage of the vesicles, we performed fluorescence microscopy experiments on the leakage of the water-soluble fluorescent dye calcein. We detected only very low leakage (less than 10% in 30 min) in the case of uncharged membranes (mixtureE see Figure 4 and Figure 5). This fact may indicate very low protein binding to the GUV membrane in the absence of anionic lipids. Although we did not observe any changes in the GUV shape for lipid mixtures A and B (Figure 3), we observed slow calcein leakage from the GUVs with these lipid compositions during 30 min for mixture B with 0 mol% cholesterol and during 25 min for mixture A with 15 mol% cholesterol. This means that the E protein at low cholesterol formed small membrane pores, which were permeable to calcein. However, the rate of leakage was rather slow to induce changes in the GUV shape. The presence of 30 mol% cholesterol in the GUV resulted in a significant acceleration of the calcein leakage (Figure 6). For 40 mol% cholesterol, the initial leakage rate was almost 30 times higher than for 30 mol% cholesterol (Figure 6). These results, together with the significant deformations of the GUV shape (Figure 2), suggest possible assembly of highly permeable membrane channels by the E protein that differ structurally from those formed for 0 or 15 mol% cholesterol.

Neither for the H3 peptide (see Figure 6) nor for the control protein SUMO-1 (Figure S4B) did we detect any significant leakage (it was less than 10%). This means that the H3 peptide did not demonstrate any membrane activity. Additionally, we proved that SUMO-1, used in our construct with the E protein, had no effect on the calcein leakage.

### 3.3. Patch-Clamp Experiments Reveal Different Types of E Protein-Induced Membrane Conductance

For all charged lipid compositions studied (mixtures A–D), we observed significant leakage of calcein from the vesicles during adsorption of the E protein. However, the leakage rate varied with cholesterol content. This suggests that the SARS-CoV-2 E protein is capable of forming pores in vesicle membranes, but by different mechanisms. To investigate this phenomenon in more detail, we recorded the current through the lipid bilayer using the patch-clamp technique. A micropipette was filled with the E protein solution in working buffer 1 and placed in tight contact with the BLM. For a concentration of 1 µM of the protein, we observed ion-conducting pores of different lifetimes and different types of electrical signals.

**Figure 7:**
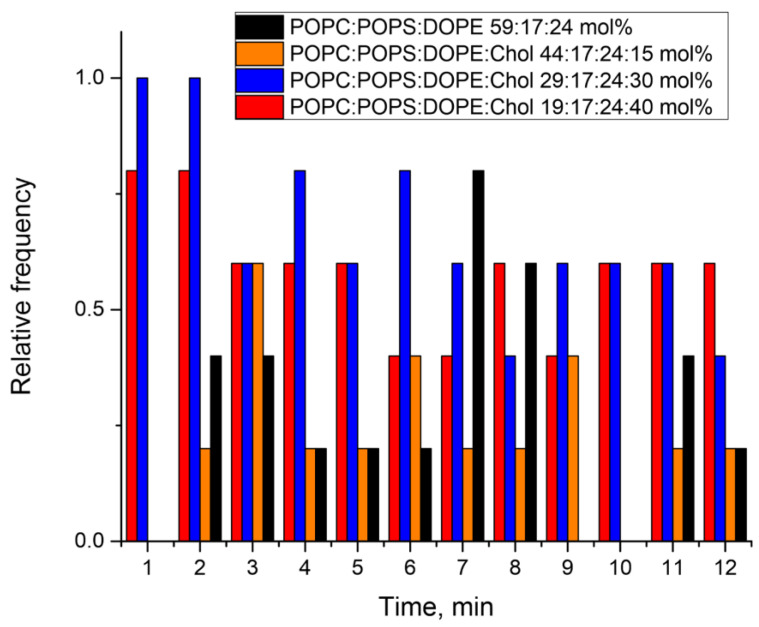
Histogram of the relative frequency of occurrence of conductance changes over the course of the experiments (12 min) for 1 µM of the E protein, summarized from 5 experiments for four lipid mixture A (orange), mixture B (black), mixture C (blue), and mixture D (red).

To analyze the total membrane conductance upon addition of the E protein to the lipid membranes of all lipid compositions (mixtures A–E), the results were presented as follows. For each min (total time 12 min), the number of recordings in which any type of signal was observed was calculated. The resulting number of experiments was then divided by the total number of experiments. The data are presented as the relative frequency of occurrence of a signal of any type (Figure 7). The most significant conductance bursts were observed for the lipid mixtures containing 30 mol% and 40 mol% cholesterol, while a weak conductance response occurred for the lipid mixtures containing 15 mol% and 0 mol% cholesterol. In the absence of negatively charged lipids in the membrane composition (mixture E), only a weak increase in the conductance was recorded in the first minute of the experiment (Figure S5).

**Figure 8:**
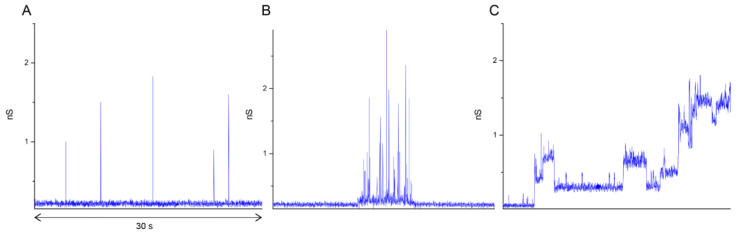
Typical conductance traces of the three types of electrical signals obtained by the patch-clamp experiments: spike (**A**), multi-level (**B**), and square-top (**C**) signals. The total duration of each record displayed is 30 s.

**Figure 9:**
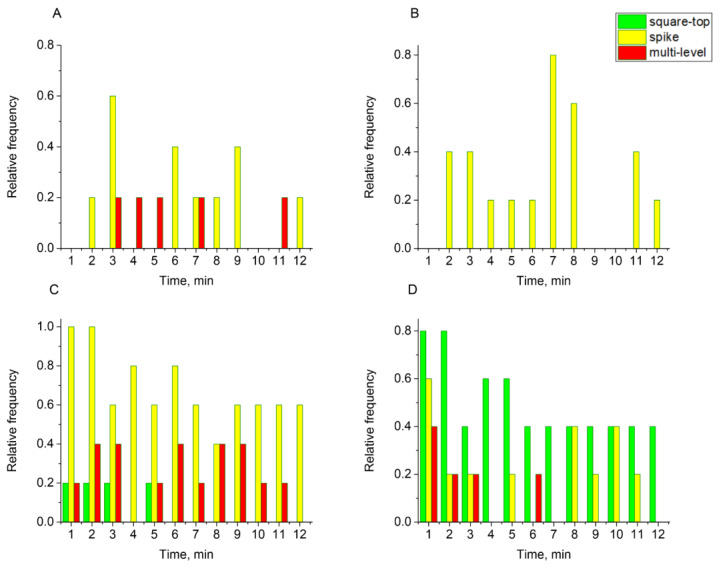
Histograms of the relative frequency of occurrence of each type of electrical signal, spike, multi-level, and square-top signals, over the course of the experiments (12 min) for 1 µM of the E protein, summarized from 5 experiments for lipid mixtures A–D (panels (**A**–**D**).

To investigate the influence of cholesterol in the lipid composition on the membrane conductance changes, we analyzed in detail the electrical signals obtained for each lipid mixture. In general, conductance signals across a lipid membrane in the presence of pore-forming molecules can be divided into three main groups based on the patch-clamp signal classification [56]: spike, multi-level, and square-top signals (see Figure 8). Spike signals correspond to the formation of very short-living pores, whereas square-top signals are associated with long-living channel-like activity as a result of the incorporation of cooperative structures of several pore-forming peptides or proteins into the membrane. With the addition of the E protein to the BLM of mixture B, we observed an appearance of spike conduction (Figure 9B). In this case, the protein behaves as an amphipathic peptide, in which the main type of conductance is only a short-living defect corresponding to the possible passage of the individual peptide through a lipidic pore in the membrane [59]. The absence of other types of conductance indicates the inability of the E protein to form stable channels for the given lipid composition. For mixture A, containing 15 mol% cholesterol, we observed the appearance of spike conductance and a few multi-level signals (Figure 9A). For mixture C, containing 30 mol% cholesterol, we recorded a small number of square-top signals, as well as multi-level signals, indicating the possibility of the cooperative interaction of E protein molecules under these conditions (Figure 9C). However, these events were rather short-living. Only for 40 mol% cholesterol (mixture D) we did observe square-top signals that were stable for several min (Figure 9D). This suggests stable channels formed by the E protein rather than the cooperative translocation of several protein molecules.

To verify whether the presence of the H3 peptide can cause the appearance of any conductance signals, we recorded the current through the lipid bilayer from mixture D during the adsorption of 20 µM H3 peptide. The obtained results, summarized from 5 experiments of 15 min each, revealed an absence of any ion-conducting events (Figure S6). As a control, we also tested SUMO-1 for the possibility to induce membrane conductance. No signals were detected for 15 min upon addition of 1 µM SUMO-1 (Figure S7).

### 3.4. Atomic Force Microscopy Demonstrates the Ability of the E Protein to Form Semi-Transmembrane or Transmembrane Pores Depending on the Cholesterol Content of a Membrane

**Figure 10:**
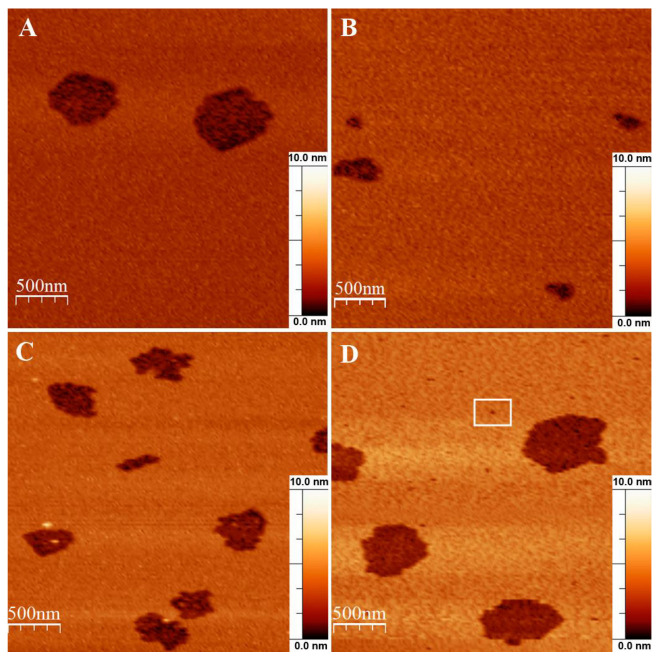
Typical AFM images of the supported lipid bilayers of lipid mixtures A–D (panels (**A**–**D**), respectively) exposed to 100 nM E protein. White frame in panel D highlights an example of the smallest defect, which is further shown at higher resolution in Figure 11. Image size is 3 × 3 μm^2^. Scale bar is 10 nm.

The results of confocal fluorescence microscopy on GUVs and patch-clamp measurements on BLMs showed that the E protein forms different types of pores or channels in the membrane depending on the cholesterol content. To investigate these structures at the molecular level, we decided to use AFM. At the beginning of each experiment, we confirmed that our supported lipid bilayers were free of any defects (Figure S8). Then, we added 100 nM E protein solution to the AFM fluid cell without further incubation in air. AFM height images of the bilayers exposed to the E protein are shown in Figure 10.

**Figure 11:**
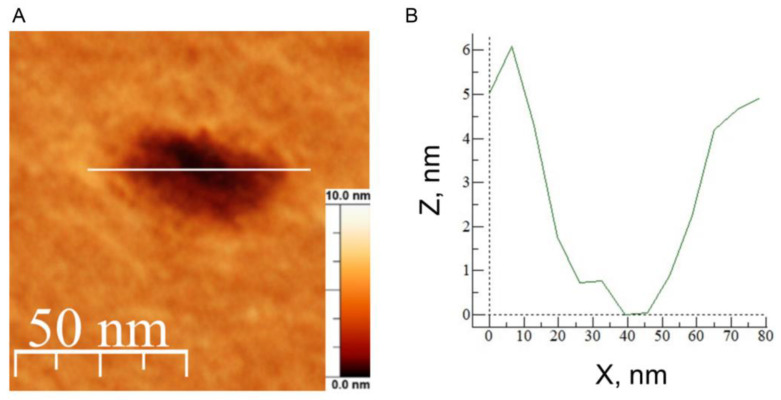
High-resolution AFM image of an example of the smallest defect, highlighted by the white frame in Figure 10D (**A**). Image size is 0.1 × 0.1 μm^2^. Scale bar is 10 nm. The profile is taken along the white line across the defect (**B**).

We found that the smallest area occupied by the defects was for mixture B without cholesterol, while for the other mixtures it increased with the cholesterol content (see Figure 10). However, the transverse size of these defects was hundreds of nm, which is unlikely to be the size of the viroporin channel. The exception was in the case of lipid mixture D, where we detected stable small defects (white frame in Figure 10D) with diameters of 20–30 nm and depths of about 4 nm (Figure 11). Since the resolution of the AFM in the *z*-axis direction reaches 0.1 nm, while in the lateral plane it is determined by the size of the cantilever tip and is not higher than several nanometers, we decided to further analyze only the depth of the defects.

**Figure 12:**
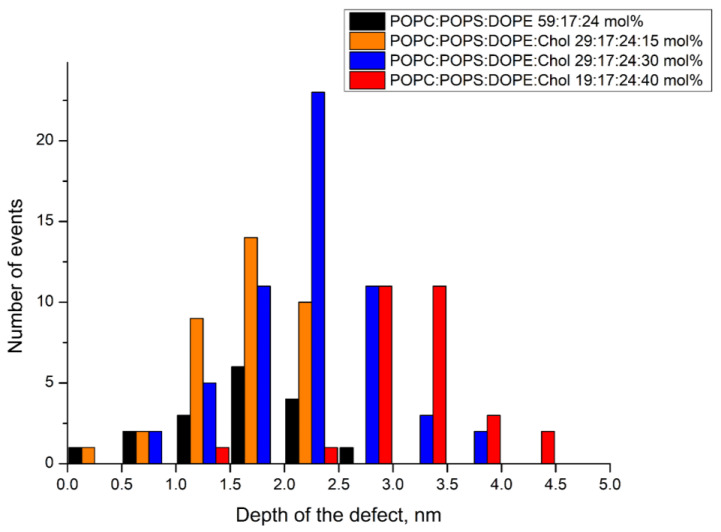
Comparison of the characteristic values of the depths of the membrane defects formed in a supported lipid bilayer upon adsorption of 100 nM E protein for lipid mixture A (orange), mixture B (black), mixture C (blue), and mixture D (red).

The most interesting thing is that the depth of these protein-induced defects also increased with the amount of cholesterol in the lipid mixture (Figure 12). For 0 and 15 mol% of cholesterol the depth was about 2 nm, while for 30 mol% cholesterol it increased up to 4 nm, and for 40 mol% cholesterol almost all defects were 4 nm in depth. The membrane thickness for the studied lipid compositions varied from 3.5 nm to 3.8 nm [60]. This means that at 40 mol% cholesterol in the membrane, we observed transmembrane pores, while at 0 mol% and 15 mol% they spanned only half of the bilayer thickness. At 30 mol% cholesterol we observed a combination of different types of defects, or pores, from semi-transmembrane to complete transmembrane ones.

We observed only very rare defects, with depths of about 1.5 nm, for the case of uncharged lipid mixture E (Figure S9), suggesting the necessity of not only cholesterol but also anionic lipids for E protein membrane binding. For the H3 peptide and SUMO-1, we did not detect any changes in the AFM images (Figures S10 and S11, respectively). As many peptides and small proteins might be invisible in AFM images, if these molecules lay barely on the membrane [61,62], we cannot prove that they did not interact with the membrane. However, we can state that both the H3 peptide and SUMO-1 do not form pores in the membrane.

## 4. Discussion

Viroporins are unique viral ion channels of up to 120 amino acids with at least one transmembrane domain and an amphipathic helix [8,32,33]. Typically, viroporins oligomerize from multiple molecules in the membranes of different cellular compartments. Despite their ion channel activity, viroporins have distinct functions at different stages of the viral life cycle [9]. Since they are produced inside the infected cell, they can interfere with cell signaling, energy production, inflammatory responses, and so on [8,36]. At the same time, viroporins of several viruses are involved in membrane deformation for virus budding and egress [36]. However, for many viroporins, their distinct role in viral pathogenesis is still elusive.

The SARS-CoV-2 E protein is a structural protein of the viral envelope typical for different coronaviruses. It is a viroporin that is crucial for the infectivity of the virion [23]. It is reported to participate in viral budding by generating membrane curvature [14,20], to initiate a cytokine storm inside the infected cell [18], and to diminish electrochemical gradients at the membranes of cell compartments [7]. At the same time, which part of the protein is responsible for its membrane activity, what is the stoichiometry of the assembled viroporin, as well as the role of the lipid environment in the functionality of the E protein are still under debate.

Here, we combined fluorescence confocal microscopy, patch-clamp experiments, and atomic force microscopy to show the lipid-dependent behavior of the E protein at the structural and functional level. First, we demonstrated that this protein binds very slightly to the uncharged membranes, in line with previous studies, which have shown a very low and non-selective conductance of the E protein viroporin for pure zwitterionic membranes [7,63]. The presence of negatively charged POPS allows the formation of ion and calcein conducting pores in the membrane. Our patch-clamp measurements showed that these pores are very short-living spike conducting events (Figure 8A and Figure 9B), which are typically associated with lipidic pores or peptide translocation through the membrane [47,59].

Indeed, the E protein comprises a long juxtamembrane region at the C-terminal part, which is believed to be amphipathic [20] (Figure 1). We took the longest structured part of this region, the H3 α-helix, and synthesized the corresponding peptide. However, we did not detect any membrane activity of this part of the E protein. Because we used a water-soluble construct of the E protein with SUMO-1 at the N-terminus (Figure 1), we doubt that the N-terminal part of the protein could translocate across the membrane in approximately 10 ms. Additionally, our control experiments demonstrated an absence of membrane deformations by SUMO-1 used in the construct with the E protein for all membrane compositions studied, in contrast with the isolated SUMO-1 protein. Therefore, the observed spike conductance in the presence of negatively charged lipids suggested the formation of transient lipidic pores near the E protein molecules. These pores can form in the vicinity of just two amphipathic molecules because they deform the membrane [47]. Possibly, our result correlates with the NMR and mass-spectrometry studies demonstrating a dimer of the transmembrane domains of the E protein in charged membranes without cholesterol [45] or detergent micelles [46]: these dimers are able to form lipidic pores by deforming the membrane, while the dimer is not enough to form a stable ion channel.

Surprisingly, AFM experiments for lipid mixture B demonstrated the formation of semi-transmembrane defects spanning a depth of about 1.8 nm (Figure 9B and Figure 10, black), meaning the removal of lipids from only one membrane monolayer, and having a lateral size of hundreds of nanometers. The formation of such defects has been reported for several amphipathic peptides like α-synuclein, and β_L_- and γ-crystallin [64,65]. The authors describe this effect as the formation of peptide–lipid micelles from the contacting membrane leaflet. The question arises: how do these structures relate to the short-living lipidic pores observed by patch-clamp measurements? In fact, the whole E protein with the transmembrane domain and the polar C-terminal part could be treated as an amphipathic molecule. The reported structure of the protein dimer [45,46] supports this idea. Insertion of the amphipathic molecule into the lipid bilayer induces an asymmetry in membrane tension between its leaflets [66]. As a result, the lipid bilayer should deform towards the immersed molecule. This imbalance relaxes through the formation of pores in a membrane. However, in the case of the supported lipid bilayer with one leaflet bound to the mica surface by Van der Waals and electrostatic interactions, this is impossible, and only the contact monolayer should relieve the elastic stress. Thus, it forms micelles, while protein molecules cover hydrophobic tails of the distant monolayer.

Recently, we have shown that the same defects are formed in the presence of amyloid precursor protein (APP), which, like the E protein, has transmembrane and juxtamembrane helices [67]. Moreover, in [21] the authors show that the C-terminal part of the E protein adopts a β-sheet conformation, which only changes to amphipathic α-helical in the presence of membrane curvature. This is also a good analogue for APP. In our work [67], we have demonstrated that the depth of the APP immersion into the membrane depends on the membrane cholesterol content. Thus, we decided to study the effect of cholesterol on the membrane activity of the E protein. We found that increasing the cholesterol content led to the appearance of more long-living multi-level conductance signals (Figure 8B and Figure 9A), while at 30 and 40 mol% cholesterol we observed a significant increase in the calcein leakage (Figure 5), as well as the induction of square-top conductance signals (Figure 8B,C and Figure 9C,D), which are usually associated with the formation of stable ion channels in the membrane, like in the case of alamethicin [59,68]. Our AFM data suggest the presence of stable channel-like transmembrane defects in the case of high cholesterol content in a membrane (Figure 11). Simultaneously, we observed deformations of the GUVs for 30 and 40 mol% cholesterol after addition of the E protein (Figure 2C,D). Molecular dynamics simulations suggest that this protein is able to induce membrane curvature [20]. Several viroporins are reported to have similar membrane-deforming activity and even the ability to form DMVs at the endoplasmic reticulum of the cell [37,38,39,40]. However, other studies demonstrate that the E protein alone keeps the membrane flat [22]. Our results showed no evidence of DMVs under the action of the E protein for any of the lipid mixtures studied. The observed GUV deformations are clearly related to the significantly increased membrane leakage, which results in the loss of the spherical shape of the vesicles. In AFM experiments, we detected that the high amount of cholesterol in the membrane increases the depth of the pores formed by the E protein, up to the full thickness of the membrane, which is about 3.8 nm [60]. Together with the patch-clamp results, we could hypothesize that after some threshold of about 30 mol% cholesterol in the membrane, we observed the formation of stable viroporins by the E protein. Possibly, they are related to the formation of pentameric channels in the membrane [50]. It is noteworthy that the limits of the AFM lateral resolution of several nanometers would not allow the detection of small lipid pores if they existed in the supported lipid bilayer. Nevertheless, the depth of the defects represents membrane structures with a much higher resolution of about 0.1 nm. Therefore, we based our conclusions only on this parameter.

**Figure 13:**
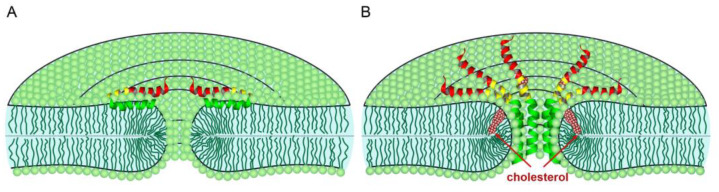
Schematic representation of the SARS-CoV-2 E protein activity depending on the cholesterol content: lipidic pore induced by the E protein dimer in a membrane with low (15%) or zero cholesterol (**A**); E protein pentameric channel in the membrane stabilized by high amount of cholesterol (30–40 mol%) (**B**).

In Figure 13A, we schematically show a possible transient lipidic pore induced by the E-protein dimer, and in Figure 13B, the possible E-protein pentameric channel formed at 30–40 mol% cholesterol. Interestingly, these amounts of cholesterol are not typical for ERGIC [20,50], where they reach 15 mol%, but typical for the plasma membrane [51]. Therefore, the formation of such channels could occur in the plasma membrane of an infected cell or in some other cell compartments.

## 5. Conclusions

In the present study, we demonstrated both structurally, using high-resolution AFM, and functionally, by fluorescence confocal microscopy and patch-clamp measurements, that the E protein forms different structures in the membrane depending on its lipid composition. While the protein does not bind to uncharged membranes, its interaction with anionic lipid bilayers depends on the amount of cholesterol. At zero or low cholesterol content, the E protein molecule behaves like an amphipathic moiety, inducing GUV leakage and the appearance of small membrane pores, presumably mainly lipidic. We hypothesize that under these conditions the oligomerization state of the E protein is a dimer. However, above a threshold of about 30 mol% cholesterol in the membrane, this protein forms stable long-living channels that might be associated with its pentameric oligomer. Because different cellular membranes comprise different amounts of cholesterol, and the E protein has been found in many of them in the infected cell, we can conclude that the diversity of its reported functions might be the result of specific protein–lipid interactions. In fact, this diversity is an interplay between the membrane cholesterol content and the ability of the E protein to form oligomers with different structures and functional roles.

## Data Availability

All the data are available on demand.

## Supplementary Materials

The following supporting information can be downloaded at: https://www.mdpi.com/article/10.3390/biom14091061/s1, Figure S1: Mass spectrum and HPLC of the synthetic H3 peptide of the E protein; Figure S2: Bright field image of the micropipette placed in the vicinity to the selected GUV; Figure S3: GUV stability test in the absence of the proteins; Figure S4: Influence of SUMO-1 on the GUV shape and the calcein leakage; Figure S5: Typical kinetics of the membrane conductance upon addition of 1 μM E protein to the lipid mixture E; Figure S6. Typical kinetics of the membrane conductance upon addition of 20 μM H3 peptide to the lipid mixture D; Figure S7: Typical kinetics of the membrane conductance upon addition of 1 μM SUMO-1 to the lipid mixture D; Figure S8: Typical AFM images of the supported lipid bilayer of the lipid mixtures A–E; Figure S9: Typical AFM image of the supported lipid bilayer of the lipid mixture E exposed to 100 nM E protein; Figure S10: Typical AFM image of the supported lipid bilayer of the lipid mixture D exposed to 100 nM H3 peptide; Figure S11: Typical AFM image of the supported lipid bilayer of the lipid mixture D exposed to 100 nM SUMO-1.
