# Supplementary material for: Membrane Activity and Viroporin Assembly for the SARS-CoV-2 E Protein Are Regulated by Cholesterol
Source: Biomolecules. 2024 Aug 26;14(9):1061. doi: 10.3390/biom14091061 (PMC11430671; doi:10.3390/biom14091061)
Supplement: Supplementary file 1 [file biomolecules-14-01061-s001.zip › biomolecules-3135893-supplementary.pdf]

Supplementary Material

Membrane activity and viroporin assembly for the E protein from SARS-CoV-2 are regulated by cholesterol

Marta V. Volovik[1], Zaret G. Denieva[1], Polina K. Gifer[1], Maria A. Rakitina[2], Oleg V. Batishchev[1,*]

1 Laboratory of Bioelectrochemistry, A.N. Frumkin Institute of Physical Chemistry and Electrochemistry, Russian Academy of Sciences, 31/4 Leninskiy prospekt, 119071, Moscow, Russia; marta.volovik@phystech.edu (M.V.V.);
2 zaret03@mail.ru (Z.G.D.); gifer.pk@phystech.edu (P.K.G.)
N.I. Pirogov Russian National Research Medical University of the Ministry of Health of the Russian Federation, 1 Ostrovityanova street, 117997, Moscow, Russia

*Correspondence: olegbati@gmail.com

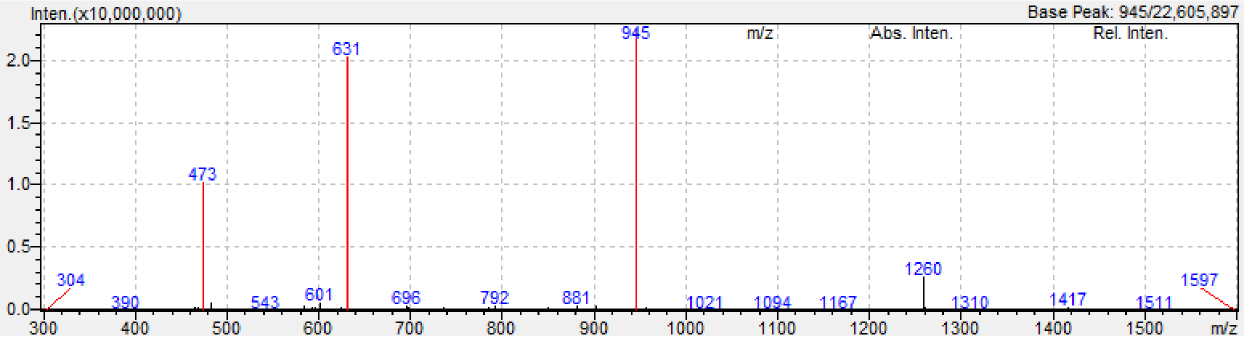

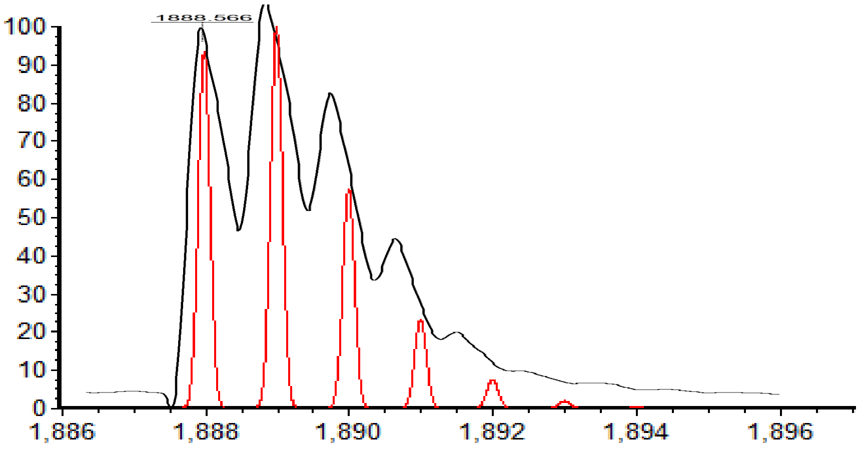

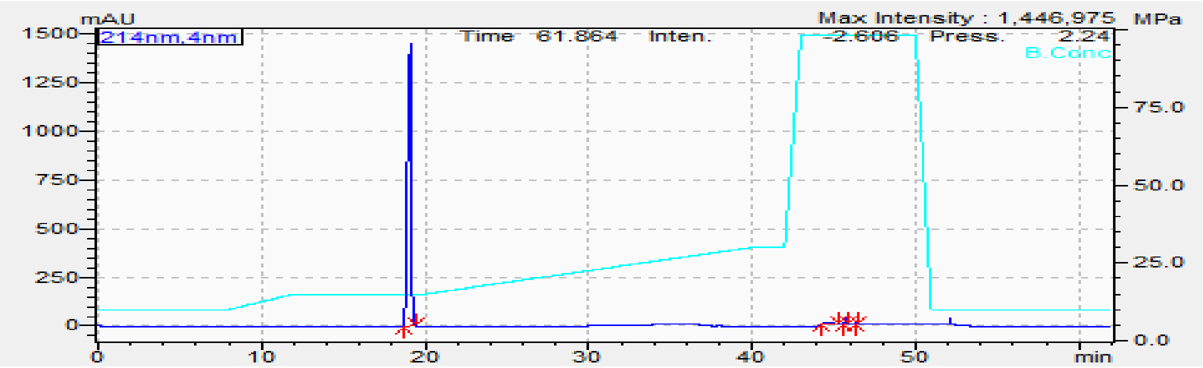

	Ret. Time (min)	Area	Area (%)
Peak 1	19.1	22993698	97.83
Peak 2	44.67	231756	0.99
Peak 3	45.79	186604	0.79
Peak 4	46.43	91014	0.39

Figure S1. Mass spectrum (upper panel) and HPLC (lower panel) of the synthetic H3 peptide of the E protein. The theoretical mass of the peptide is 1889 Da. The sequence of the peptide is NH_2-KPSFYVYSRVKNLNSS-$CONH_2$. The peaks highlighted in red refer to charged ions of the peptide: 473 m/z for (+4); 631 m/z for (+3); 983 m/z for (+2). The theoretical (red line) and experimental (black line) mass spectra of a molecular ion calculated as 1888.566.

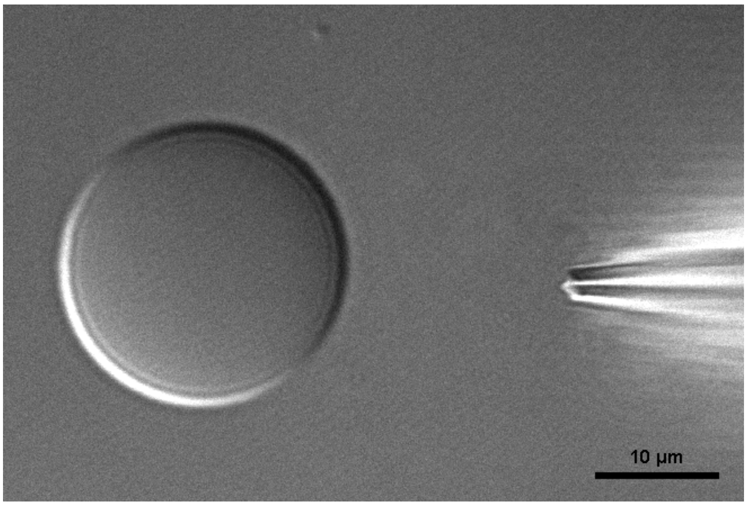

Figure S2. The typical bright field image of the micropipette placed in the vicinity to the selected GUV.

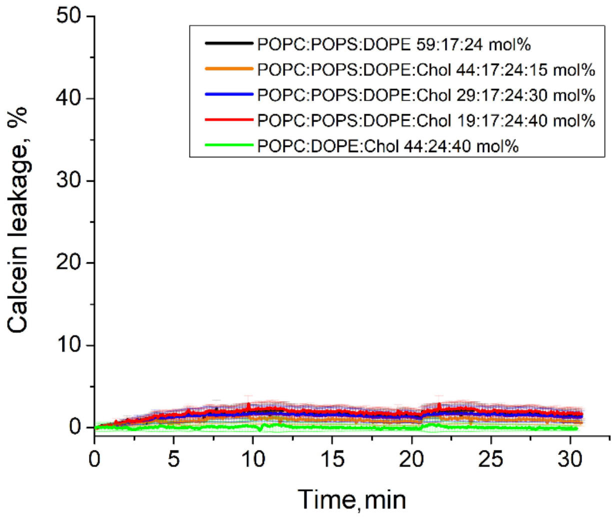

Figure S3. GUVs stability test in the absence of the proteins.

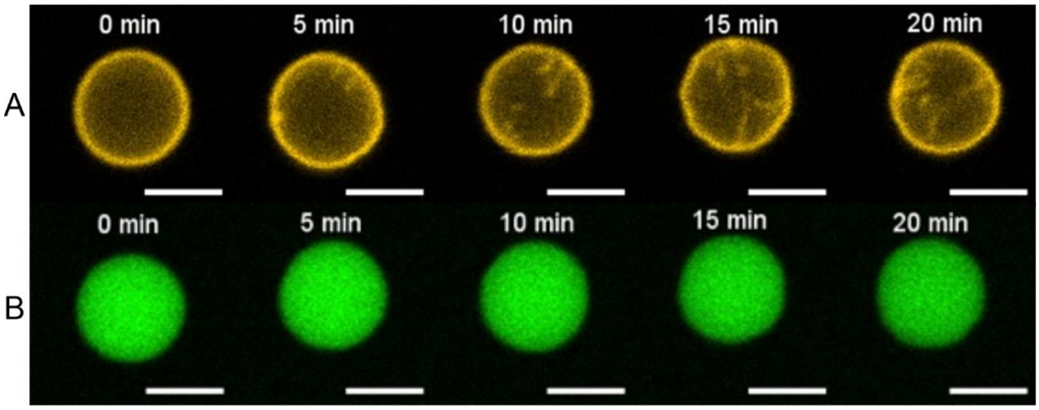

Figure S4. Influence of isolated SUMO-1 on the GUV shape and the calcein leakage. A. Typical fluorescence images of deformations of the GUV after the addition of 1 μM of SUMO-1 in the vicinity of the GUV from lipid mixture D. The numbers above each GUV indicate the time in minutes from the start of the protein or peptide addition. B. Typical fluorescence images of the calcein leakage from the GUVs from lipid mixture D after the addition of 1 μM SUMO-1. The numbers above each GUV indicate the time in minutes from the start of the protein addition. Scalebar is 10 μm.

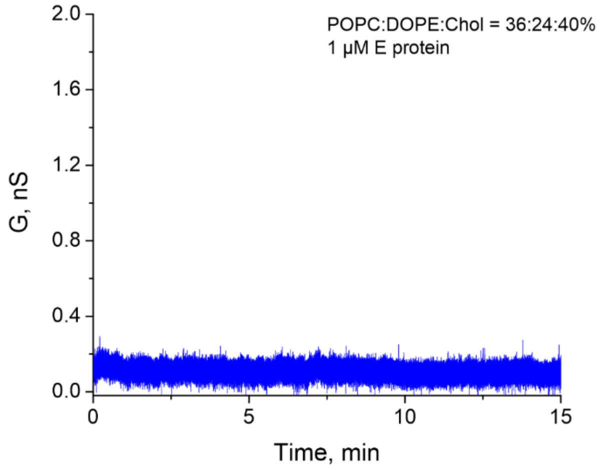

Figure S5. Typical kinetics of the membrane conductance upon addition of 1 μM E protein to lipid mixture E.

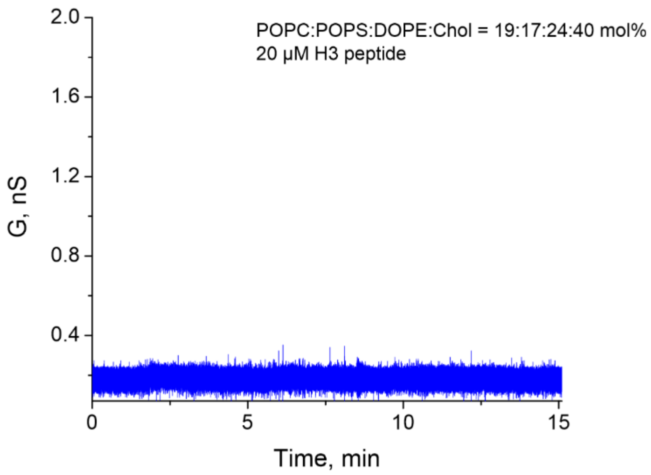

Figure S6. Typical kinetics of the membrane conductance upon addition of 20 μM H3 peptide to lipid mixture D.

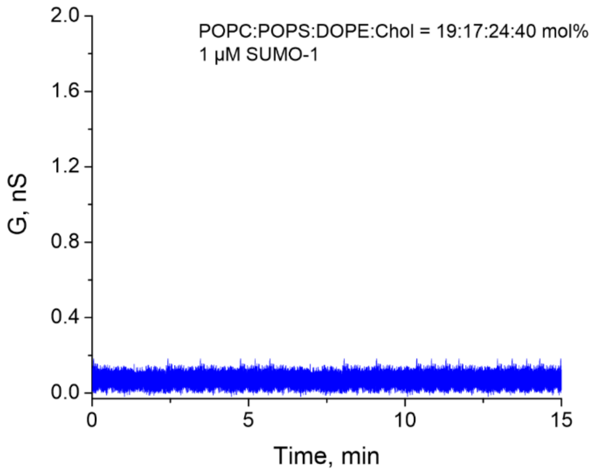

Figure S7. Typical kinetics of the membrane conductance upon addition of 1 μM SUMO-1 to lipid mixture D.

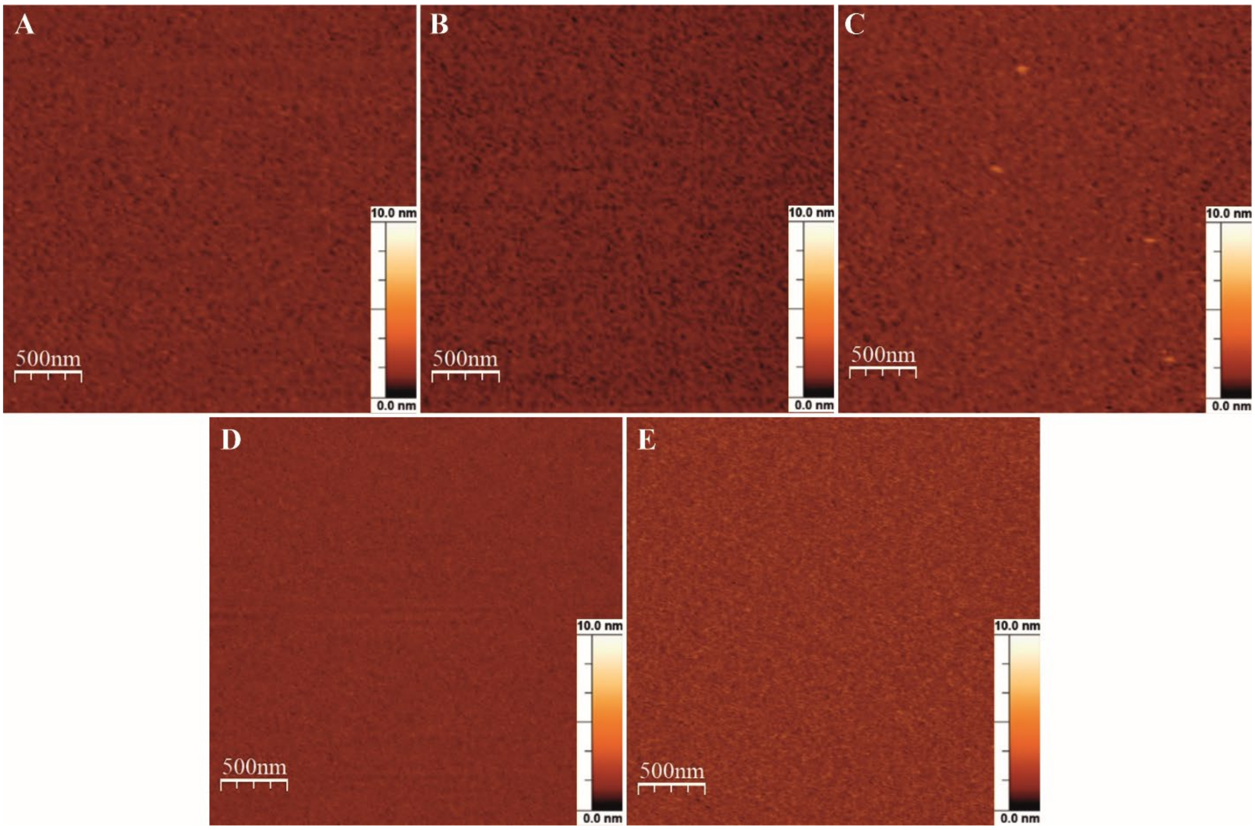

Figure S8. Typical AFM images of the supported lipid bilayer of the lipid mixtures A-E. Image size is 3 × 3 μm^2. Scale bar is 10 nm.

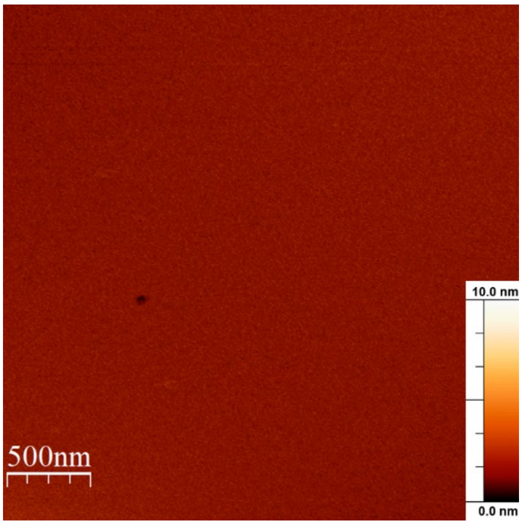

Figure S9. Typical AFM image of the supported lipid bilayer of the lipid mixture E exposed to 100 nM E protein. Image size is 3 × 3 μm^2. Scale bar is 10 nm.

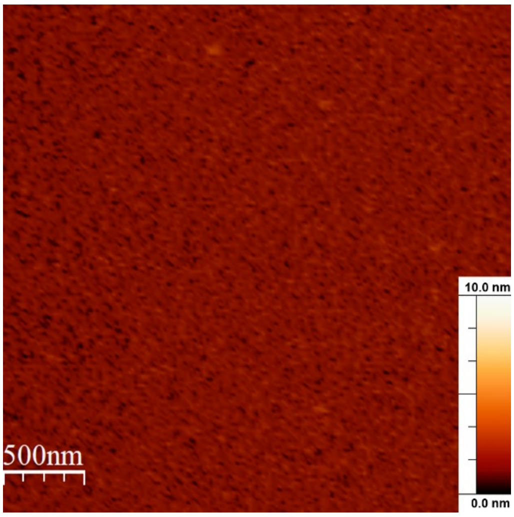

Figure S10. Typical AFM image of the supported lipid bilayer of the lipid mixture D exposed to 100 nM H3 peptide. Image size is 3 × 3 μm². Scale bar is 10 nm.

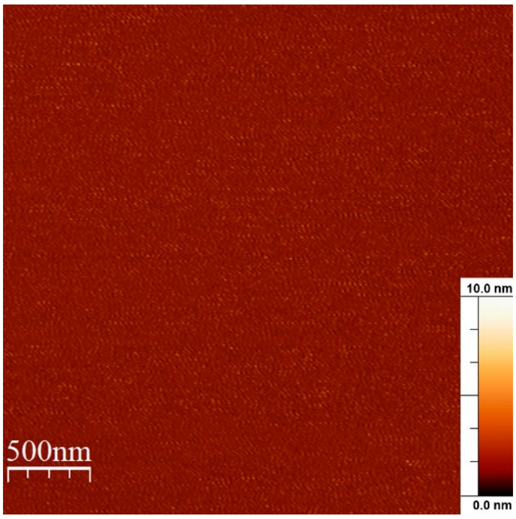

Figure S11. Typical AFM image of the supported lipid bilayer of the lipid mixture D exposed to 100 nM SUMO-1. Image size is 3 × 3 μm². Scale bar is 10 nm.
